# Selection for Test-Day Milk Yield and Thermotolerance in Brazilian Holstein Cattle

**DOI:** 10.3390/ani11010128

**Published:** 2021-01-08

**Authors:** Renata Negri, Ignacio Aguilar, Giovani Luis Feltes, Jaime Araújo Cobuci

**Affiliations:** 1 Department of Animal Science, Federal University of Rio Grande do Sul, Porto Alegre 91540-000, Brazil; feltesgiovani@gmail.com; 2 Department of Animal Breeding, Instituto Nacional de Investigación Agropecuaria, Montevideo 11100, Uruguay; iaguilar@inia.org.uy

**Keywords:** animal resilience, genotype-by-environment interaction, longitudinal data

## Abstract

**Simple Summary:**

Interest in selection for milk yield and thermotolerance in cattle has grown, since heat stress has caused great losses in milk yield. However, few studies on how to carry out concurrent selection are available. Milk yield was analyzed by traditional methods, including heat stress indicators, in genetic evaluation. The results showed that the best sires for milk yield are not the best for heat tolerance, and only a small proportion of individuals have the aptitude for joint selection. Despite a small population fraction allowed for joint selection, sufficient genetic variability for selecting more resilient sires was found, which promoted concomitant genetic gains in milk yield and thermotolerance.

**Abstract:**

Intense selection for milk yield has increased environmental sensitivity in animals, and currently, heat stress is an expensive problem in dairy farming. The objectives were to identify the best model for characterizing environmental sensitivity in Holstein cattle, using the test-day milk yield (TDMY) combined with the temperature–humidity index (THI), and identify sires genetically superior for heat-stress (HS) tolerance and milk yield, through random regression. The data comprised 94,549 TDMYs of 11,294 first-parity Holstein cows in Brazil, collected from 1997 to 2013. The yield data were fitted to Legendre orthogonal polynomials, linear splines and the Wilmink function. The THI (the average of two days before the dairy control) was used as an environmental gradient. An animal model that fitted production using a Legendre polynomials of quartic order for the days in milk and quadratic equations for the THI presented a better quality of fit (Akaike’s information criterion (AIC) and Bayesian information criterion (BIC)). The Spearman correlation coefficient of greatest impact was 0.54, between the top 1% for TDMY and top 1% for HS. Only 9% of the sires showed plasticity and an aptitude for joint selection. Thus, despite the small population fraction allowed for joint selection, sufficient genetic variability for selecting more resilient sires was found, which promoted concomitant genetic gains in milk yield and thermotolerance.

## 1. Introduction

Dairy breeding programs have traditionally focused on selection for milk yield. This intense selection has increased sensitivity to environmental changes in animals. Climate and variability in climate have negatively affect milk yield due to impacts on metabolic efficiency and immune responses [1]. Currently, heat stress is an expensive problem in dairy farming.

Garcia et al. [2] observed a 21% milk yield loss in a commercial herd of Holstein cows in southern Brazil caused by heat stress. According to Pegorer et al. [3], approximately 60% of dairy farms in the world are in heat-stress environments. Heat stress decreases milk yield by 30% to 40% [4], which represents approximately 600 to 900 kg of milk per lactation per cow [5], and can exceed 1300 kg of milk per cow [6]. The impact of heat stress on the dairy cattle industry resulted in an economic loss of USD 900 million in 2012 [4], and the estimated loss in 2014 was USD 1.2 billion for the US dairy sector [7].

According to Sigdel et al. [8] and Ansari-Mahyari et al. [9], a possible strategy for reducing the effects of heat stress on dairy cattle is the selection of genetically more thermotolerant animals. However, it requires methodology that allows for the identification and subsequent selection of animals according to specific regions and climates.

Brazil has a diverse climate, from warm and dry/humid to cold and humid climates, due to a wide variation in longitude, latitude, and altitude in its territory and the effects of coastal, continental vegetation [10]. In addition, it is affected by seasonal factors, which influence the management of herds. In warmer conditions, animals tend to be kept on pasture (to use the grass cycle), while in colder periods, animals are semi-confined and supplemented with silage [11]. Thus, each region of the country is more suitable and productive for specific dairy cattle genotypes, which requires a combined selection for heat tolerance and milk yield for each region.

Different thermotolerances are found between and within dairy cattle breeds. The selection of animals within a breed is an alternative when crossbreeding is not feasible. Physical sizes, metabolic rates and productive levels have been associated with thermotolerance [12].

Ravagnolo and Misztal [13] described a method to identify the most resilient animals regarding heat tolerance, proposing a random regression method that quantifies the heat stress level based on climate information (the temperature–humidity index) on the test day. This methodology allows for the detection of the presence of genotype-by-environment interactions (G × Es), and the modeling of animal performance as a function of the environmental gradient.

The evaluation of decreases in milk yield per THI-unit increase from a determined threshold is a method for predicting the relationship between production and climate conditions [5]. The THI is a bioclimatic index commonly used to determine heat stress in cattle [14]. Diurnal temperature variation (DTV) also has potential for use as an environmental indicator of heat stress [15].

Several hypothetical models and fitting equations have been proposed to estimate the effect of heat stress. A combination of methods allows the identification of the best methodology for explaining the variation in genetic and non-genetic components within an environmental gradient. This enables critical genetic analysis and the reduction of the negative effects of heat stress on dairy cattle performance and impacts the dairy production chain.

In this context, the objective of this study was to identify the best model for characterizing environmental sensitivity in Holstein cattle in Brazil, using the test-day milk yield combined with temperature–humidity index data from public weather stations, and identify sires genetically superior in terms of heat tolerance and milk yield through random regression models.

## 2. Materials and Methods

### 2.1. Climate Data

Bioclimatic data from 18 weather stations (representing 86 municipalities) were obtained from the Instituto Nacional de Meteorologia. The stations were located within 60 km of the evaluated farms. The temperature–humidity index (THI) was calculated according to the equation described by the National Research Council—NRC [16]:

THI = [(1.8 × DBT + 32) − (0.55 − (0.0055 × RH) × (1.8 × DBT − 26))](1)

where DBT is the dry bulb temperature (°C) and RH is the relative humidity (%). The THI was found to range from 50 to 95.

**Figure 1:**
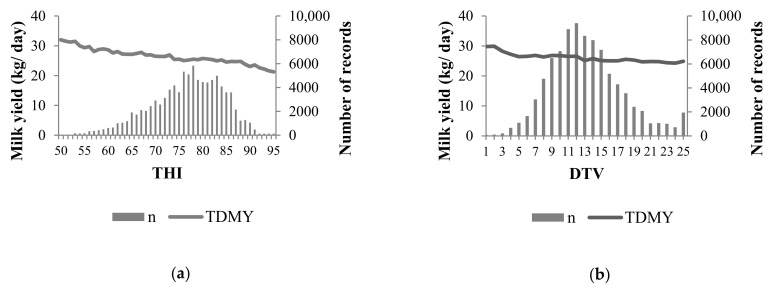
Number of records (n) and means of test-day milk yield (TDMY) per temperature–humidity index (THI) (**a**) and diurnal temperature variation (DTV) (**b**) in Brazilian Holstein cattle.

The diurnal temperature variation (DTV) was calculated by the difference between the daily maximum and minimum temperatures (°C). The DTV was found to range from 2 to 25. The numbers of test-day milk yield records by the THI and DTV are shown in Figure 1.

The THI and DTV daily averages used were calculated based on averages of two days (one and two days before the test-day milk yield records), as described by Negri et al. [17].

### 2.2. Data

The test-day milk yield (TDMY) data, provided by the Associação de Criadores de Gado Holandês de Minas Gerais (ACGHMG), consisted of records of Holstein cows at first lactation in the state of Minas Gerais, Brazil (19°55′ S–43°57′ W), from 1996 to 2015.

Records of animals with ages at calving <18 or >48 months, days in milk (DIM) <5 or >305 days, and milk yields <4 or >44.8 kg were excluded from the data set. Only healthy animals with at least four TDMY records during lactation were used for analysis. The minimum size of each contemporary group (CG; based on the herd, years and months of the TDMY records) was three animals. Records of daughters of sires with at least one daughter in at least three herds were included in evaluation.

Considering these criteria, a total of 94,549 TDMY records from 11,294 first lactations of Holstein cows, with a mean test-day milk yield of 25.81 kg, from 129 herds, collected from 1997 to 2013, were analyzed. This database was fitted to all the evaluated models. The pedigree file included 32,409 animals. The structure of the dataset after editing is shown in Table 1.

### 2.3. Models

Five models (M1 to M5) and three fitting equations were used to analyze the TDMY records: the Wilmink parametric function (WL) [18], linear splines (LS) [19], and Legendre orthogonal polynomial (LP) [20]. Additive genetic and permanent environmental (co)variance functions were regressed to the THI, DTV and DIM, according to the models described below.

Model 1 (M1): The contemporary group, milking frequency (two and three times a day), variable *t* (described below the models), and DIM with 60 classes (every five units of DIM was considered a class: DIM 5 to 10 = class 1, DIM 11 to 15 = class 2… DIM 300 and 305 = class 60) were fixed effects. The fixed curve (described below the models), additive genetic and permanent environmental functions were regressed to the THI, using the LP (4th order), LS (4 knots) and WL.

Model 2 (M2): The contemporary group, milking frequency and variable *t* were fixed effects. The fixed curve, additive genetic and permanent environmental functions were regressed: the DIM using the LP (4th order), and to the THI using the LP (2nd order); to the DIM using LS (4 knots) and to the THI using LS (3 knots); to the DIM and THI using the WL.

Model 3 (M3): The contemporary group, milking frequency and variable *t* were fixed effects. The fixed curve, additive genetic and permanent environmental functions were regressed: to the DIM using the LP (4th order), and to the THI and DTV using the LP (2nd order); to the DIM using LS (4 knots), and to the THI and DTV using LS (3 knots); to the DIM, THI and DTV using the WL.

Model 4 (M4): The contemporary group, milking frequency, variable *t* and DTV (with 5 classes: DTV 2 to 6 = class 1, DTV 7 to 11 = class 2… DTV 22 to 25 = class 5) were fixed effects. The fixed curve, additive genetic and permanent environmental functions were regressed: to the DIM using the LP (4th order), and to the THI using the LP (2nd order); to the DIM using LS (4 knots), and to the THI using LS (3 knots); to the DIM and THI using the WL.

Model 5 (M5): The contemporary group, milking frequency, variable *t* and DIM with 60 classes (every five units of DIM was considered a class: DIM 5 to 10 = class 1, DIM 11 to 15 = class 2… DIM 300 and 305 = class 60) were fixed effects. The fixed curve, additive genetic and permanent environmental functions were regressed: to the THI and DTV using the LP (2nd order); to the THI and DTV using LS (3 knots); to the THI and DTV using the WL.

A dummy variable *t* was defined to estimate the decreases in milk yield caused by heat stress (HS). The threshold for HS used was a THI of 74, based on Negri et al. [17]. Therefore,

*if* THI ≤ 74, *t* = 0 (no heat stress); *else if THI > 74 then t* = THI − 74. (2)

The fixed curves considered in all the models were defined by the age classes—1 (18 to 25 months), 2 (26 to 27 months), 3 (28 to 29 months) and 4 (30 to 48 months)—combined with calving season subclasses—1 (rainy: October to March) and 2 (dry: April to September)—totaling eight fixed curves. The residual variance was considered homogeneous in all the models (Table 2).

### 2.4. Analysis of Models

Random regression models (RRM) were used for analysis. Henderson’s mixed model equations [21] for RRM can be described as follows:

(3) $$\left[\begin{array}{ccc} X^{\prime }R^{-1}X & X^{\prime }R^{-1}Z & X^{\prime }R^{-1}W\\ Z^{\prime }R^{-1}X & Z^{\prime }R^{-1}Z+A^{-1}⊗G_{0}^{-1} & Z^{\prime }R^{-1}W\\ W^{\prime }R^{-1}X & W^{\prime }R^{-1}Z & W^{\prime }R^{-1}W+I⊗P_{0}^{-1} \end{array}\right]\left[\begin{array}{c} \hat{b}\\ \hat{a}\\ \hat{p} \end{array}\right]=\left[\begin{array}{c} X^{\prime }R^{-1}y\\ Z^{\prime }R^{-1}y\\ W^{\prime }R^{-1}y \end{array}\right]$$

where **y** is the vector of observations; **X**, **Z** and **W** are the incidence matrices for the fixed effects(**b**), additive genetic random regression coefficients (**a**), and permanent environmental random regression coefficients (**p**), respectively; **A** is the additive genetic numerator relationship matrix based on pedigree information; and **I** is an identity matrix. **G**_0_ and **P**_0_ are the (co)variance matrices of the additive genetic and permanent environmental random regression coefficients, respectively, and **R** is the (co)variance matrix of the residual.

All the genetic analyses were performed with an animal model, using the REMLF90 program [22]. Considering the REML estimation method, the model assumptions can be described as:

(4) $$\left[\begin{array}{c} y\\ a\\ p\\ e \end{array}\right]\sim N\left(\left[\begin{array}{c} X\beta \\ 0\\ 0\\ 0 \end{array}\right];\left[\begin{array}{cccc} ZGZ^{\prime }+WPW^{\prime }+R & ZG & WP & R\\ & A⊗G_{0} & \phi & \phi \\ & & I⊗P_{0} & \phi \\ Sim. & & & R \end{array}\right]\right)$$

where **e** is the vector of the residuals, and all the other terms were previously defined. The genetic (**Σ**) and environmental (**Φ**) (co)variance matrices for time points can be obtained as follows (assuming the same function for factors):

(5) $$\Sigma =TG_{0}T^{\prime }\;\mathrm{and}\;\Phi =TP_{0}T^{\prime }$$

where **T** is a matrix of independent covariates for all time points (DIM, THI or DTV) associated with the model and function used.

The quality of fit was evaluated considering non-nested models and penalties, according to the number of parameters to be estimated. The following criteria were used: the maximum likelihood estimation (−2logL), Akaike’s information criterion (AIC = −2logL + 2p, where *p* is the number of parameters in the model), and Schwarz’s Bayesian information criterion (BIC = −2logL + p log (λ), where log (λ) is the natural logarithm of the sample size (or dimension of *y*) and *p* is the number of parameters in the model). The BIC is more rigid than AIC. The model with the lowest value for both criteria was the one with the best fit.

The estimated breeding value (EBV) of an animal *i* obtained with M4 was computed using DIM (EBV_TDMY) and environmental gradient (THI values, EBV_HS) information, according to the equation

(6) $$EBV_{l}^{j,k}=\varphi_{(j)k}{â}_{i}^{\prime }$$

where ${â}_{i}^{\prime }$ is the vector of the estimated additive genetic values for the orthogonal regression coefficients of animal *i* (coefficients corresponding to DIM and THI) and $\varphi_{(j)k}$ is a vector of the orthogonal coefficients evaluated in THI *j* and DIM *k*.

The top 1% and 5% dairy Holstein sires (with at least 20 daughters) for EBV_TDMY and EBV_HS were sampled to represent the resilience of the animals, considering the DIM and THI scale (i.e., reaction norms).

## 3. Results

### 3.1. Loss in Milk Yield and Adjustment of Models

Considering only the THI, approximately 31% of all the TDMY records were obtained under heat-comfort conditions (<THI 74) and 69% were under HS conditions. The data show that the milk yield tended to decrease as the THI was increasing. The mean and standard deviation of the TDMY were 28.57 ± 1.8 kg for THI < 74, and 23.95 ± 1.9 kg for THI > 74.

Approximately 46% of all the TDMY records were obtained at DTV within heat comfort conditions (<DTV 13), and 54% were in HS conditions. The mean and standard deviation of the TDMY records were 27.33 ± 1.3 kg for DTV < 13 and 24.94 ± 0.4 kg for DTV > 13. The phenotypic value tended to decrease as the DTV increased. However, slope is more representative for assessing the THI effect.

**Figure 2:**
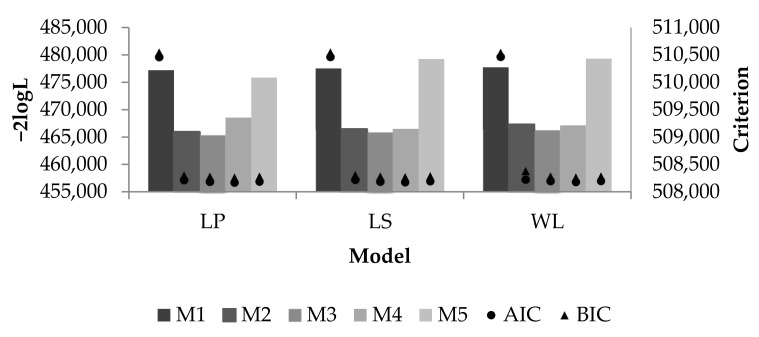
Estimates of maximum of likelihood function (−2logL), Akaike’s information criterion (AIC) and Schwarz’s Bayesian information criterion (BIC) according to evaluation model and random effects adjustment equation of test-day milk yield (TDMY) in Brazilian Holsteins.

Five models that used the LP to fit fixed and random curves showed better quality of fit (Figure 2). The best model according to the information criteria (AIC and BIC) was M4, with AIC = 508,170 and BIC = 508,264, which regresses data to the DIM and THI, and included the DTV as a fixed effect. Thus, in addition to the model minimizing the Kullback–Leibler divergence (related to missing information), the probability of fit of the true model is maximized.

AIC includes the complexity and predicted ability of the data to fit the model, and is linked to the BIC due to the probability function. However, the BIC penalizes models more because of the number of parameters. Thus, the quality of fit (AIC and BIC) indicates the same model, confirming that the chosen model is the most appropriate.

### 3.2. Heritability

**Figure 3:**
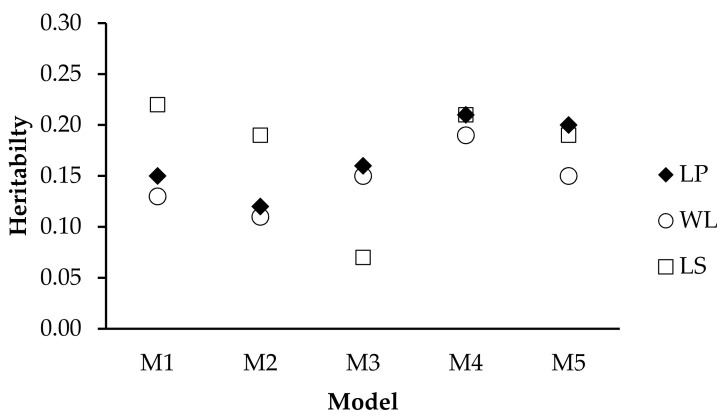
Estimated average heritability for test-day milk yield (TDMY) according to temperature–humidity index (THI) according to model and using Legendre polynomials (LP), linear splines (LS) and Wilmink (WL) in Brazilian Holsteins.

The overall heritability estimate for the TDMY regressed to the THI ranged from 0.15 to 0.21 when using the LP equation (Figure 3), while for the WL, it ranged from 0.11 to 0.19, and when using LS, it varied from 0.07 to 0.22.

**Figure 4:**
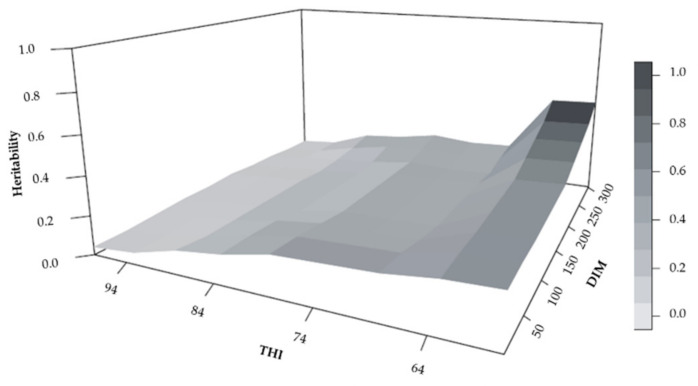
Estimated heritability for test-day milk yield, TDMY, according to temperature–humidity index (THI) and days in milk (DIM) according to model 4 (M4) using Legendre polynomials.

The heritability estimated as a function of lactation in M4 using the LP (best fit) weighted by the THI and DIM, after the THI threshold (THI = 74), showed a decrease (Figure 4). The environmental variation increased, and the additive genetic variation decreased, directly interfering with the heritability of the trait. Thus, the highest selection responses can be expected for the thermal-comfort range. The extrapolation of estimates close to the beginning (DIM 5) and end (DIM 305) of lactation was due to the low number of registered TDMYs.

### 3.3. Genotype-by-Environment Interaction

**Figure 5:**
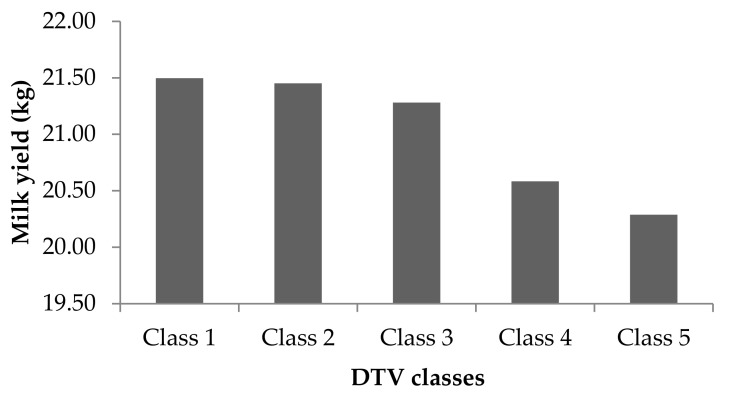
Solution of fixed effect (BLUE) of temperature variation (DTV) according to classes considered in the model 4 (M4) model using Legendre polynomials.

The fixed effects solution (BLUE) for DTV shows that an increase in the variation temperature is reflected in a decrease in milk yield (Figure 5). The fixed effect considered in model that regresses to the DIM and THI (M4) better explains the behavior of the lactation curve, since the temperature variation needs to be nested at some thermal reference point in order to define the magnitude and importance of its effect. Thus, the use of DTV should always be nested in a heat-stress indicator, in order to anchor the inferred heat comfort.

Classes: DTV 2 to 6 = class 1; DTV 7 to 11 = class 2; DTV 12 to 16 = class 3; DTV 17 to 21 = class 4; DTV 22 to 25 = class 5.

**Figure 6:**
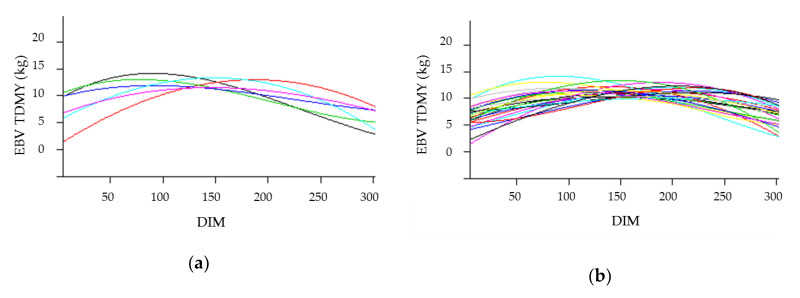

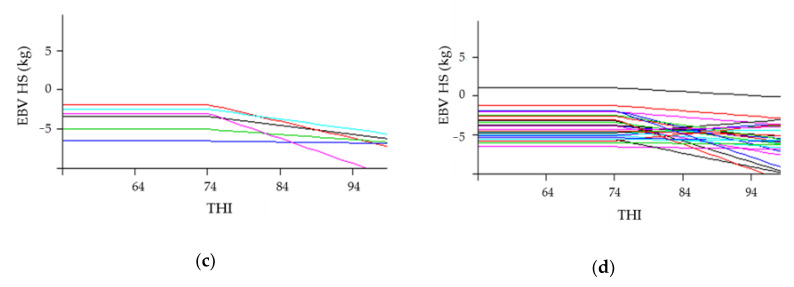
Estimated breeding value gradient (i.e., reaction norm model) for best sires: top 1% (**a**) and top 5% (**b**) for test-day milk yield (EBV_TDMY) and for tolerance to heat stress (EBV_HS) of the same sires (**c**,**d**).

The existence of additive genetic variability for the slope of the environmental gradient indicated the presence of G×E interaction (Figure 6). Some of the best sires (EBV_TDMY) (Figure 6a,b) had interesting EBV_HS (Figure 6c,d).

Approximately 9% of the sires were resilient to changes in the THI, considering their EBV for TDMY and HS tolerance; however, approximately 30% showed probable plasticity. This allows the selection and formation of a resilient lineage with good productivity.

**Figure 7:**
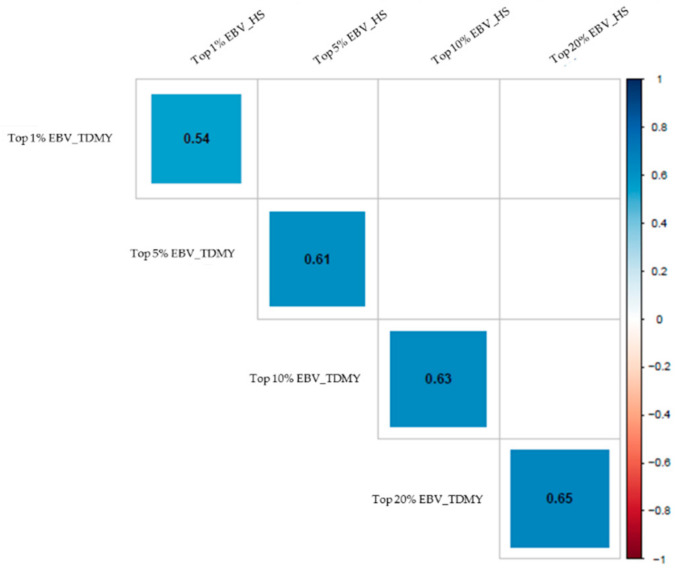
Spearman rank correlation coefficients for top 1%, top 5%, top 10% and top 20% best sires (estimated breeding values—EBVs) regarding test-day milk yield (TDMY) and heat stress (HS), according to model 4 (M4) using Legendre polynomials. The colors inside the squares indicate the magnitudes and directions of the associations.

The reranking of sires was confirmed by the Spearman rank correlation coefficients, comparing the genetic evaluation for TDMY and thermotolerance (Figure 7). Therefore, the Holstein sires with the best EBV_TDMY records in favorable environments may not be the best under EBV_HS conditions. Moreover, the impact of reranking is higher when the selection pressure is the strongest (Top 1%).

The database, in which 90% of the records were measured under heat-stress conditions, showed that the traditional model (which does not consider heat stress) is inefficient for estimating the EBV when animals are under heat stress. Thus, considering two situations, when sires are considered equally in heat comfort (THI = 74) and in heat stress (THI = 84), sires with less than 40 daughters are the most penalized, and their EBVs are underestimated (Table 3).

The effect of the genotype-by-environment interaction is minimized and the traditional methodology is not biased when a sire has more than 101 daughters in several herds. However, some breeders intensively use high EBV_TDMY, and others use them less often. This unequal contribution to adaptive values makes the estimates biased.

## 4. Discussion

Most dairy cows in Brazil are kept in open barns and in grazing systems [23,24,25]. Minas Gerais has three predominant climate types—subtropical at altitude, subtropical with dry winters, and tropical with dry winters—according to the Köppen—Geiger classification. Thus, environmental factors such as the THI and DTV have a direct impact on cow productivity. Lactating dairy cows must be in favorable environments because stresses negatively affect cow maintenance, milk yield, growth, the preservation of body condition (health) and reproduction. 

Climate changes may increase heat-stress levels in dairy cows; thus, these environmental phenomena, which frequently cause droughts, heat waves, storms and floods, should be considered [26]. Increases in climate variability have negatively impacted livestock production, especially dairy farming [15,27]. Temperatures in tropical regions increased by 0.1 to 0.3 °C per decade between 1951 and 2000 because of increases in greenhouse gases [26], and the variations in temperatures have increased by 0.7 to 0.8 °C because of the El Niño Southern Oscillation (ENSO) over the past century [28]. However, the selection of animals for milk yield has not considered animals’ tolerance to heat stress, as shown in the present study.

These changes justify and foster research that evaluates the effects of climate variables on animal production, mainly regarding genetic breeding, in which the effects are cumulative and long term. In addition, the use of heat stress indicators collected from public weather stations, rather than directly from farms, has been widely explored for inclusion in genetic evaluation. According to Lee et al. [29], the evaluation should not be affected by this substitution, because the use of contemporary groups compensates for effects at the farm, management, nutrition and technological levels.

Legendre polynomials are more widely used for these evaluations [30,31,32,33]. Despite the complexity of these assessments, better results can be obtained when considering the two-day average of climate variables for test-day milk yield, including the DTV as a fixed effect in the model and regressing data to the THI and DIM. 

A low number of observations of extremes of lactation may affect the prediction coefficients of functions fitted through random regression [34]. The same database was fitted to LP, WL and LS functions, and the LP was the more robust model, considering possible biases caused by the low number of observations at the ends of the gradient.

The sooner HS is detected, the greater the chances of keeping more resilient animals in production and, consequently, the more productive they are in different heat conditions. According to Aguilar et al. [35], the genetic variance of heat stress for milk yield increases significantly from the first lactation. Thus, cows become more sensitive to heat stress as the number of parities is increased. Thus, detecting susceptibility to heat stress in the first lactation allows the prediction of losses in subsequent lactations.

Brügemann et al. [36] emphasize that the effect of heat stress can suppress the expression of animals’ genetic potential, and reported that a random regression model showed a trend of higher heritability in the THI range corresponding to the comfort zone of cows, as found in the present study. Thus, the selection of these superior environments can contribute to accurate genetic differentiation among candidates for selection.

The use of random regression to detect G × E fits a variance–covariance structure of repeated measures along a gradient for traits such as the TDMY [37]. Similarly, the model proposed by Kolmodin et al. [34], using covariance functions, is a good G × E indicator.

Selecting sires only by EBV_TDMY, disregarding the herd rearing system, technological level and bioclimatic conditions of a region, may compromise the genetic gain of herds. Moreover, investments in high-merit genetic material from animals evaluated in highly technological environments should not be recommended for farmers in environments with low technological investments. 

An ideal curve would be a high and constant EBV_TDMY under different THIs; however, few animals presented this desired profile. Thus, considering the needs for breeding of each herd is important for more quicky increasing the TDMY or tolerance to HS. Each sire has a thermotolerance slope limit, as shown by the difference between the curves, which allows the better targeting of sires for different uses, according to farmers’ demands.

The results shown in Figure 6 denote the possibility of using individual random slopes as a selection criterion for resilience (animals that withstand variations that can cause heat stress). Animals with a subtle slope are less sensitive to environmental variation. The results indicate high genetic variations in sires within different environments. 

This high variability indicates that the apparent genetic merit of sires for milk yield may change depending on the environment, generating great concern about genetic evaluation and the choice of sires, which can be affected by this dependence. Phenotypic and genetic parameters are dependent on the population and environment, and can have different magnitudes, resulting in heterogenous variations. Thus, changes in variances and the covariance would promote changes in important parameters, such as heritability, repeatability and correlations, which may result in incorrect choices of selection methods adopted in a breeding program.

According to Santana et al. [31], the flattening of the slope is particularly important for countries with a hot climate when focusing on selecting animals with high production levels and tolerant to heat stress. In tropical climate regions, the effects of environmental variations can be addressed by different methods of genetic evaluation. Thus, some applicable options can be used to simultaneously select animals for TDMY and HS *in loco*: the creation of a selection index for the simultaneous selection and targeting of mating to develop a tolerant lineage. However, it requires attention to avoid estimation biases. Negri et al. [17] pointed out the importance of correcting test-day milk yield data using heat-stress indicators and reported significant increases in estimates of repeatability and the reranking of sires, especially for sires with fewer daughters.

## 5. Conclusions

The existence of genetic variation for sensitivity to heat stress allows for the selection of genetically resilient animals.

The most adequate selection methodology for improving heat tolerance without decreasing productivity includes diurnal temperature variation as a fixed effect and regresses data to the temperature–humidity index and days in milk.

Legendre polynomials should be used to ensure better predictions of the estimated breeding value and determine the genetic effect of heat stress through random regression models.

Antagonism between the test-day milk yield and heat stress was confirmed, and enabled the use of heat tolerance as a selection criterion for improving animal thermotolerance and productivity simultaneously. The development of selection indexes weighted by technological levels or sire summaries that include information on the environmental gradient could be viable solutions. The selection and development of more thermotolerant lineages is a more attractive option than the development of selection indexes.

## Figures and Tables

**Table 1:** Overall traits of first-parity Brazilian Holstein cattle (SD in parentheses).

Item	Statistics
Number of test-day records	94,549
Number of animals with records	11,294
Number of animals in pedigree file	32,409
Number of dams in pedigree file	8639
Number of sires in pedigree file	641
Number of contemporary groups	5257
Number of herds	129
Mean test-day milk (kg)	25.81 (7.21)
Mean records/animal	8.37

**Table 2:** Model layout.

Models		Fixed Effects				Regressor		
	Contemporary Group	Milking Frequency	Variable t	DIM	DTV	DIM	THI	DTV
M1	*	*	*	*	-	-	○○○○	-
	*	*	*	*	-	-	+ + + +	-
	*	*	*	*	-	-	◊ ◊ ◊	-
M2	*	*	*	-	-	○○○○	○○	-
	*	*	*	-	-	+ + + +	+ + +	-
	*	*	*	-	-	◊ ◊ ◊	◊ ◊ ◊	-
M3	*	*	*	-	-	○○○○	○○	○○
	*	*	*	-	-	+ + + +	+ + +	+ + +
	*	*	*	-	-	◊ ◊ ◊	◊ ◊ ◊	◊ ◊ ◊
M4	*	*	*	-	*	○○○○	○○	-
	*	*	*	-	*	+ + + +	+ + +	-
	*	*	*	-	*	◊ ◊ ◊	◊ ◊ ◊	-
M5	*	*	*	*	-	-	○○	○○
	*	*	*	*	-	-	+ + +	+ + +
	*	*	*	*	-	-	◊ ◊ ◊	◊ ◊ ◊

DIM: Days in milk; DTV: Diurnal temperature variation; THI: Temperature–humidity index; * Considered; - not considered; ○○ Legendre orthogonal polynomial LP (2nd order); ○○○○ LP (4th order); + + + Linear splines LS (3 knots); + + + + LS (4 knots); ◊◊◊ Wilmink parametric function WL.

**Table 3:** Efficiency in estimating breeding value for test-day milk yield (EBV_TDMY) of Brazilian Holstein cattle under conditions of homeostasis and heat stress.

N Daughters	Sires	EBV_TDMY	
		THI 74	THI 84
>101	13	100%	91%
51 to 100	43	100%	84%
41 to 50	12	100%	84%
31 to 40	23	100%	75%
21 to 30	59	100%	74%
11 to 20	127	100%	73%
<10	364	100%	71%

## Data Availability

Restrictions apply to the availability of these data. Data was obtained from Associação de Criadores de Gado Holandês de Minas Gerais (ACGHMG).
